# Exploring the thalamus: a crucial hub for brain function and communication in patients with bulimia nervosa

**DOI:** 10.1186/s40337-023-00933-6

**Published:** 2023-11-20

**Authors:** Jiani Wang, Guowei Wu, Miao Wang, Weihua Li, Yiling Wang, Xiaodan Ren, Xuan Wei, Zhenghan Yang, Zhanjiang Li, Zhenchang Wang, Qian Chen, Peng Zhang, Lirong Tang

**Affiliations:** 1 Department of Radiology, Beijing Friendship Hospital, Capital Medical University, No. 95 Yong An Road, Xicheng District, Beijing, China; 2 CAS Key Laboratory of Behavioral Science, Institute of Psychology, Chinese Academy of Sciences, Beijing, China; 3 Chinese Institute for Brain Research, Beijing, China; 4 Beijing Anding Hospital, Capital Medical University, No. 5 Ankang Hutong, Xicheng District, Beijing, China; 5 The National Clinical Research Center for Mental Disorders and Beijing Key Laboratory of Mental Disorders, Beijing, China

**Keywords:** Bulimia nervosa, Thalamus, fMRI, Neural activity, Functional connectivity, Eating behavior

## Abstract

**Background:**

Bulimia nervosa (BN) is an eating disorder characterized by recurrent binge eating and compensatory behaviors. The thalamus plays a crucial role in the neural circuitry related to eating behavior and needs to be further explored in BN.

**Methods:**

In this study, 49 BN patients and 44 healthy controls (HCs) were recruited. We applied the fractional amplitude of low-frequency fluctuation to investigate regional brain activity in the thalamus and functional connectivity (FC) to examine the synchronization of activity between thalamic subregions and other brain regions in both groups. All results underwent false discovery rate (*p* < 0.05, FDR correction) correction. Pearson correlation analysis was performed to assess the relationship between the patients’ abnormal clinical performance and the thalamic alterations (*p* < 0.05, FDR correction).

**Results:**

We found no significant differences in neural activity between BN patients and HCs in the sixteen thalamic subregions. However, compared to the HCs, the individuals with BN showed decreased FC between the thalamic subregions and several regions, including the bilateral prefrontal cortex, right inferior parietal lobule, right supplementary motor area, right insula, cingulate gyrus and vermis. Additionally, BN patients showed increased FC between the thalamic subregions and visual association regions, primary sensorimotor cortex, and left cerebellum. These altered FC patterns in the thalamus were found to be correlated with clinical variables (the frequency of binge eating/purging per week and external eating behavior scale scores) in the BN group. All results have passed FDR correction.

**Conclusions:**

Our study provides evidence that there is disrupted FC between thalamic subregions and other brain regions in BN patients during resting state. These regions are primarily located within the frontoparietal network, default mode network, somatosensory, and visual network. These findings elucidate the neural activity characteristics underlying BN and suggest that thalamic subregions have potential as targets for future neuromodulation interventions.

**Supplementary Information:**

The online version contains supplementary material available at 10.1186/s40337-023-00933-6.

## Abstract (plain language summary)

The high recurrence rate of bulimia nervosa (BN) poses a clinical challenge, and thus, it is crucial to improve the characterization and identification of brain functional abnormalities as direct targets for novel therapies. To investigate the neural circuitry associated with BN, the thalamus is a critical node since it serves as a higher-order relay point in the cortico-thalamo-cortical information pathway. Our findings reveal that altered functional connectivity (FC) between thalamic nuclei and other brain regions is evident throughout the whole brain, particularly within the frontoparietal network, default mode network, somatosensory, and visual network. These changes in FC are significantly associated with disordered eating behavior and the severity of illness in BN patients. Therefore, these findings help identify the neural mechanisms underlying disordered eating behavior and BN severity and suggest potential targets for future neuromodulation interventions.

**Supplementary Information:**

The online version contains supplementary material available at 10.1186/s40337-023-00933-6.

## Background

Bulimia nervosa (BN) is an eating disorder characterized by recurrent binge eating and compensatory behaviors, and is frequently associated with severe medical complications and psychiatric comorbidities [1]. While remission occurs in up to 80% of treated patients, the 20% relapse rate still poses a clinical challenge, and the disorder is also associated with a higher rate of mortality [2]. Further improvements in the characterization and identification of brain functional abnormalities as direct targets for novel therapies are crucial [3].

Several psychopathological dimensions, such as cognitive challenges, impulsivity, attention difficulties, inability to delay reward, and negative self-evaluation regarding body image, are believed to be associated with abnormal eating behaviors in BN patients [4]. The thalamus is commonly viewed as a sensory relay station for information flow from the various sensory pathways to the cerebral cortex [5, 6]. The thalamus serves as a higher-order relay station, playing a vital role in the cortico-thalamo-cortical information pathway, with its functional activities encompassing cognition, somatosensory perception, visual processing, and more [5, 6]. Therefore, it holds the potential to serve as a critical node for investigating the neural circuitry associated with BN. While there have been few in-depth studies on the changes in thalamic neural activity in patients with BN, previous studies have identified BN-related brain functional and structural changes in the thalamus and its associated projection regions. For instance, a brain functional network analysis revealed that individuals with BN exhibit hypoconnectivity between the thalamus and the sensorimotor and unimodal visual association regions and that this connectivity is significantly associated with binge eating behavior [7]. Wang et al. [8] revealed that the thalamus exhibited higher positive resting-state functional connectivity (FC) in striatal nuclei, including the dorsal caudate, ventral striatum, and nearly all of the putamen subregions, in BN patients than in healthy controls (HCs).Subsequently, a graph-theoretical analysis revealed that individuals in the BN group exhibited reduced participation coefficients at the node level within the anterior prefrontal cortex, inferior parietal lobule, and thalamus. Moreover, these altered participation coefficients were significantly correlated with clinical variables, including the drive for thinness, bulimia, and interoceptive awareness of the patients [9]. Meanwhile, Berner et al. [10] examined the impact of age on subcortical volume in individuals with full and subthreshold BN, as well as in age-matched HCs. Their findings indicated that the shape of the left thalamus was inversely associated with age in the BN group, whereas it displayed no significant correlation with age in the HCs group. This suggests potential deviations in the developmental trajectory of this structure in individuals with BN. However, to date, the resting-state neural activity and FC with respect to the thalamus, especially its subregions, needs to be explored further in BN.

The human brainnetome atlas (HBA) is a valuable tool for understanding the organization of the human brain as it based on connectional architecture [11]. Based on the HBA, each side of the thalamus is divided into eight subregions, namely, the medial pre-frontal thalamus (mPFtha), pre-motor thalamus (mPMtha), sensory thalamus (Stha), rostral temporal thalamus (rTtha), posterior parietal thalamus (PPtha), occipital thalamus (Otha), caudal temporal thalamus (cTtha) and lateral pre-frontal thalamus (lPFtha). The fractional amplitude of low-frequency fluctuation (fALFF) analysis is a technique used to detect changes in regional signals in spontaneous brain activity. This method measures the relative contributions of low-frequency fluctuations within a specific frequency band (0.01–0.08 Hz) to the entire frequency range [12].In comparison to the amplitude of low-frequency fluctuation (ALFF) approach, the fALFF method mitigates nonspecific signal components in resting-state fMRI data, which leads to improved specificity and sensitivity in detecting spontaneous regional brain activity[12]. The aim of the present study is to utilize the fALFF method to investigate spontaneous regional brain activity in targeted thalamic subregions. Additionally, we performed a seed-based FC analysis to examine the synchronization of activity between these thalamic subregions and other eating-related brain regions that are spatially separated within the whole brain range [13].

Given the psychological traits of BN and the neural correlates uncovered by previous neuroimaging research, we hypothesized that the thalamus may show regional functional changes and exhibit abnormal FC between its regions and several brain regions associated with reward processing, cognitive control, and sensory processing in BN patients. Moreover, abnormal activity in the thalamus may be associated with disordered eating behaviors in individuals with BN.

## Methods

### Participants

The study was conducted in accordance with the Helsinki Declaration and approved by the Ethical Committee of the Beijing Friendship Hospital. All participants signed written informed consent forms prior to undergoing the scans.

The sample set consisted of 56 BN patients (2 male) and 46 HCs (4 male). Individuals with BN were recruited through hospital outpatient services, and HCs were recruited through local advertising. The diagnosis of BN was assessed via the Mini-International Neuropsychiatric Interview (MINI) 7.0.2 [14], a structured clinical interview developed according to the “Feeding and Eating Disorders” chapter of the diagnostic and statistical manual of mental disorders, fifth edition (DSM-5) [15]. Nine patients had previously been prescribed antidepressants to address symptoms related to anxiety or depression. Subsequently, they voluntarily discontinued their medication for various reasons, including symptom alleviation or inadequate treatment response. Importantly, none of the patients had been on psychotropic medications for a minimum of two months prior to undergoing the MRI scan. Nine BN patients had had anorexia nervosa (AN) in their lifetime, but they did not meet the diagnostic criteria of the DSM-5 during the prior 15 months. We excluded patients with major psychiatric disorders (except depression or anxiety), such as dissociative disorders, schizophrenia, and borderline personality disorder, to ensure that potentially confounding effects did not influence the results. We also performed professional face-to-face interviews with each HC based on the MINI 7.0.2 in the DSM-5 to exclude individuals with psychiatric disorders. All participants underwent serological tests to rule out metabolic conditions such as hyperthyroidism, diabetes and hyperlipidemia. Furthermore, participants with a history of malignant neoplastic disease, neurological disorders, intellectual disability, brain injury, pervasive developmental disorder, pregnancy, metal implants or claustrophobia were excluded. Among the patients included, one patient had a breast tumor, two had metal braces, and one had claustrophobia, which prevented them from undergoing an MRI examination. To reduce the influence of biological rhythm to the greatest extent possible, the subjects were informed to ensure that they obtained 8 h of sleep per night one week before the examination to maintain good sleep patterns and a good mental state. All participants underwent MRI examinations between 8 a.m. and 10 a.m. after fasting for at least eight hours. All participants completed a series of self-report questionnaires on the day of the scan, including the Dutch Eating Behavior Questionnaire (DEBQ) [16], which was used to assess eating behavior and considers three eating styles (33 items): emotional eating (distress-induced food intake), external eating (eating in response to food cues), and restraint eating (eating less than desired); the bulimia subscales from the eating disorder inventory-1 (EDI-1) [17]; 26-item eating attitude test (EAT) [18]; the 21-item Beck Depression Inventory-II(BDI-II) test [19]; and a 20-item Self-rating Anxiety Scale (SAS) questionnaire [20].The reliability and validity of the above scales have been verified in the Chinese population [17, 18, 21–25]. Furthermore, we recorded data based on the duration of the disease and the frequency of binge eating/purging behavior (which reflects the severity of the disease) for patients with BN.

### Data acquisition

All MRI images were acquired using a 3.0 T MRI system (Prisma, Siemens, Erlangen, Germany) and a 64-channel phased array coil. Axial T2-weighted imaging was performed to identify lesions. Resting-state functional images were obtained using a gradient-echo echo-planar imaging (EPI) sequence with the following parameters: 240 time points; 33 sections with 3.5-mm slice thickness; repetition time (TR)/echo time (TE), 2000/30 ms; flip angle (FA), 90°; field of view (FOV), 224 × 224 mm^2^; and a 64 × 64 matrix. High-spatial-resolution T1-weighted images were acquired using a three-dimensional magnetization-prepared rapid gradient-echo (3D-MPRAGE) sequence with the following parameters: 192 slices; slice thickness, 1 mm; TR/TE, 2530/2.98 ms; inversion time (TI), 1100 ms; FA, 7°; FOV, 256 × 256 mm^2^; and a 256 × 256 matrix.

Participants were instructed to remain awake and maintain steady breathing throughout the scan. Measures were taken to minimize head micromotion and noise interference, such as the use of air padding between the lateral sides of the head and earplugs. Participants were positioned in a supine position and placed head first into the MRI scanner. After the scan were completed, all participants reported being awake throughout the procedure. The total scanning time was approximately 14 min.

### Data preprocessing

Data Processing and Analysis for Brain Imaging (DPABI, http://www.restfmri.net) software was used to preprocess the functional images in the MATLAB 2013b environment with Statistical Parametric Mapping 12 (SPM12, http://www.fil.ion.ucl.ac.uk/spm). The first 10 dummy volumes were discarded for magnetic field equilibration and subject adaptation to the scanning noise. The remaining 33 interleaved acquisition volumes were realigned to a reference slice (mean TR slice). Participants with excessive head motion (> 2° rotational and > 2 mm translational movement) were excluded (5 participants in total, 3 from the BN group and 2 from the HC group). To account for confounding effects caused by head micromotion, the mean framewise displacement (FD) was calculated, and participants with FD ≥ 0.20 mm were excluded. The FD was also used as a covariate in the subsequent analyses. A total of 93 subjects were deemed acceptable for further analysis (see Table 1). The realigned individual brain images were normalized and resampled to 3 mm × 3 mm × 3 mm voxel size in Montreal Neurological Institute (MNI)-labeled space using the diffeomorphic anatomical registration through exponentiated lie algebra (DARTEL) algorithm [26]. Smoothing was performed using a 6 mm full-width half-maximum (FWHM) isotropic Gaussian kernel, and detrending was performed for each voxel. Covariates including the white matter (WM), cerebrospinal fluid (CSF), and Friston 24-parameter head motion were regressed from the time series of each voxel to reduce the effects of head movement and nonneural activity information. The images were then bandpass filtered in the 0.01–0.08 Hz range (this step was applied only to preprocess the data for the FC calculation and not for the fALFF calculation). Data artifact scrubbing was implemented by deleting the data for bad time points, which were defined as any volume with FD > 0.3 mm [27]. The number of excluded volumes per participant was minimal, and there was no difference between the two groups (t = 0.67, *p* = 0.51). The number of excluded volumes for the HC and BN groups was 18.70 ± 2.82 and 14.05 ± 2.01, respectively.

**Table 1:** Demographic and clinical data of participants

Variables	BN (n = 49)	HCs(n = 44)	Analysis	
			t/χ^2^	*p*
Race (Asian)	49	44	NA	NA
Sex (female/male)	(47/2)	(40/4)	0.313	0.576^a^
Age (y)	24.18 ± 5.88	25.43 ± 2.77	-1.285	0.202^b^
BMI (kg/m^2^)	21.05 ± 4.04	20.83 ± 2.04	0.317	0.752^b^
Education (y)	16.27 ± 2.01	16.86 ± 2.05	-1.420	0.159^b^
Mean FD	0.074 ± 0.03	0.069 ± 0.03	0.747	0.457^b^
Frequency	6.02 ± 4.42	NA	NA	NA
Duration of illness (m)	40.63 ± 47.22	NA	NA	NA
DEBQ-emotional	46.10 ± 12.52	27.91 ± 9.28	8.015	< 0.001^b^*
DEBQ-externality	34.96 ± 6.33	31.07 ± 4.33	3.488	0.001^b^*
DEBQ-restraint	38.24 ± 5.91	28.55 ± 7.25	7.018	< 0.001^b^*
EDI-BN	32.20 ± 6.87	11.57 ± 4.44	17.372	< 0.001^b^*
EAT	42.82 ± 10.79	12.95 ± 9.08	14.487	< 0.001^b^*
BDI	23.16 ± 8.77	3.57 ± 3.56	14.372	< 0.001^b^*
SAS	54.50 ± 11.85	33.43 ± 6.91	10.604	< 0.001^b^*

Data are presented as the mean ± standard deviation, *Statistical significance *P* < 0.05

*BN* bulimia nervosa, *HCs* healthy controls, *y* years, *m* months, *BMI* body mass index, *FD* frame-wise displacement, *Frequency* the frequency of binge eating/purging per week, *DEBQ* Dutch Eating Behavior Questionnaire, *EDI* Eating Disorders Inventory, *EAT* Eating Attitude Test, *BDI* Beck Depression Inventory, *SAS* Self-Anxiety Scale

^a^Chi-squared test

^b^Two-sample *t* tests

The structural images were preprocessed using the Computational Anatomy Toolbox 12 (CAT12: http://dbm.neuro.uni-jena.de/cat/) in the SPM12 software in MATLAB. The structural images were segmented into gray matter (GM), WM, and CSF using the unified segmentation protocol to calculate the tissue volumes. The GM segments were normalized according to the MNI template using the DARTEL algorithm and smoothed with a 6-mm FWHM isotropic Gaussian kernel [28]. To account for the potential effects of brain volume changes on the fMRI results [29], the normalized GM volume maps were included as voxelwise covariates in the subsequent functional data analyses.

### Definition of the thalamus and its subregions

**Fig. 1:**
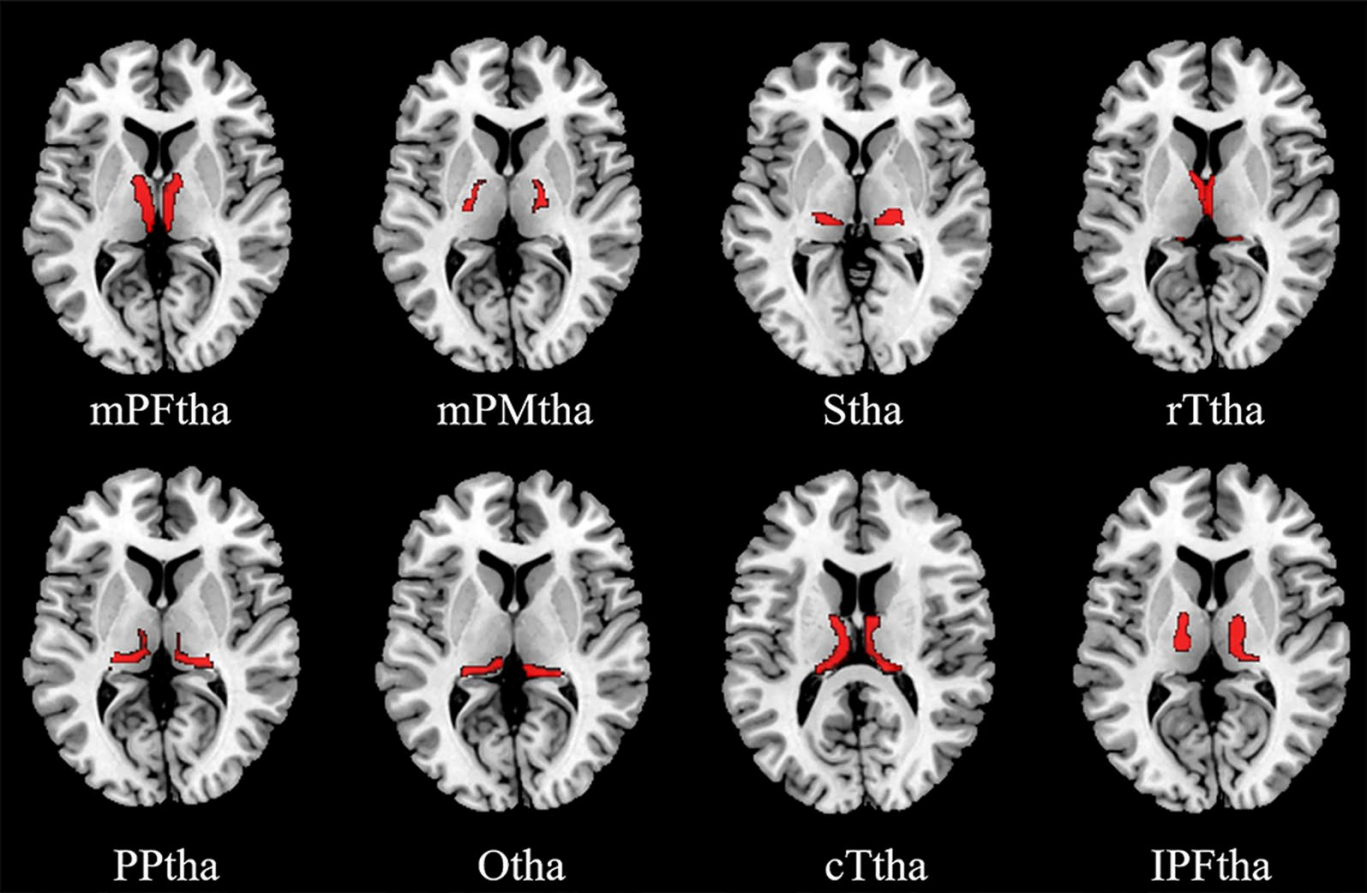
Definition of thalamic subregions based on the human brainnetome atlas. mPFtha, medial pre-frontal thalamus; mPMtha, pre-motor thalamus; Stha, sensory thalamus; rTtha, rostral temporal thalamus; PPtha, posterior parietal thalamus; Otha, occipital thalamus; cTtha, caudal temporal thalamus; lPFtha, lateral pre-frontal thalamus

The thalamus was defined based on the HBA [11], and the thalamus was further divided in sixteen subregions on the bilateral sides. Please refer to the Additional file 1 for details. This atlas was applied to all participant fALFF and FC maps by spatially normalizing all individual maps to the atlas template space (Fig. 1).

### fALFF calculation

We utilized the DPABI toolbox to calculate the fALFF, which can be used to measure the intrinsic functional architecture of local oscillation power in spontaneous brain activity [12]. The fALFF was determined by dividing the sum of the amplitude values in the low frequency range of 0.01–0.08 Hz by the sum of the amplitudes in the entire detectable power spectrum range of 0–0.25 Hz [12]. To normalize the data, the fALFF map for each voxel was divided by the mean global value of each participant.

### Voxelwise FC analysis

FC calculations were performed in a seed-to-voxel manner using the DPABI toolbox. The sixteen thalamic subregions were utilized as seed points. The reference time series for each seed point was obtained by averaging the time series of all voxels for each seed point, and their correlations with the rest of the brain were computed [13]. This procedure generated sixteen thalamus FC maps (eight per hemisphere) for each participant. The correlation coefficient maps were normalized by a Fisher r-to-z transformation.

### Statistical analysis

The demographic and clinical data differences between the two groups were analyzed using SPSS version 26.0 (IBM Corp., Armonk, New York, USA). The normality of continuous variables was assessed using Kolmogorov‒Smirnov (Lilliefors) tests. For normally distributed variables, two-sample *t* tests were used to examine group effects; otherwise, Wilcoxon rank-sum tests or Kruskal‒Wallis tests were used. Categorical variables were assessed using chi-square tests. Statistical significance was set at *p* values < 0.05.

**Fig. 2:**
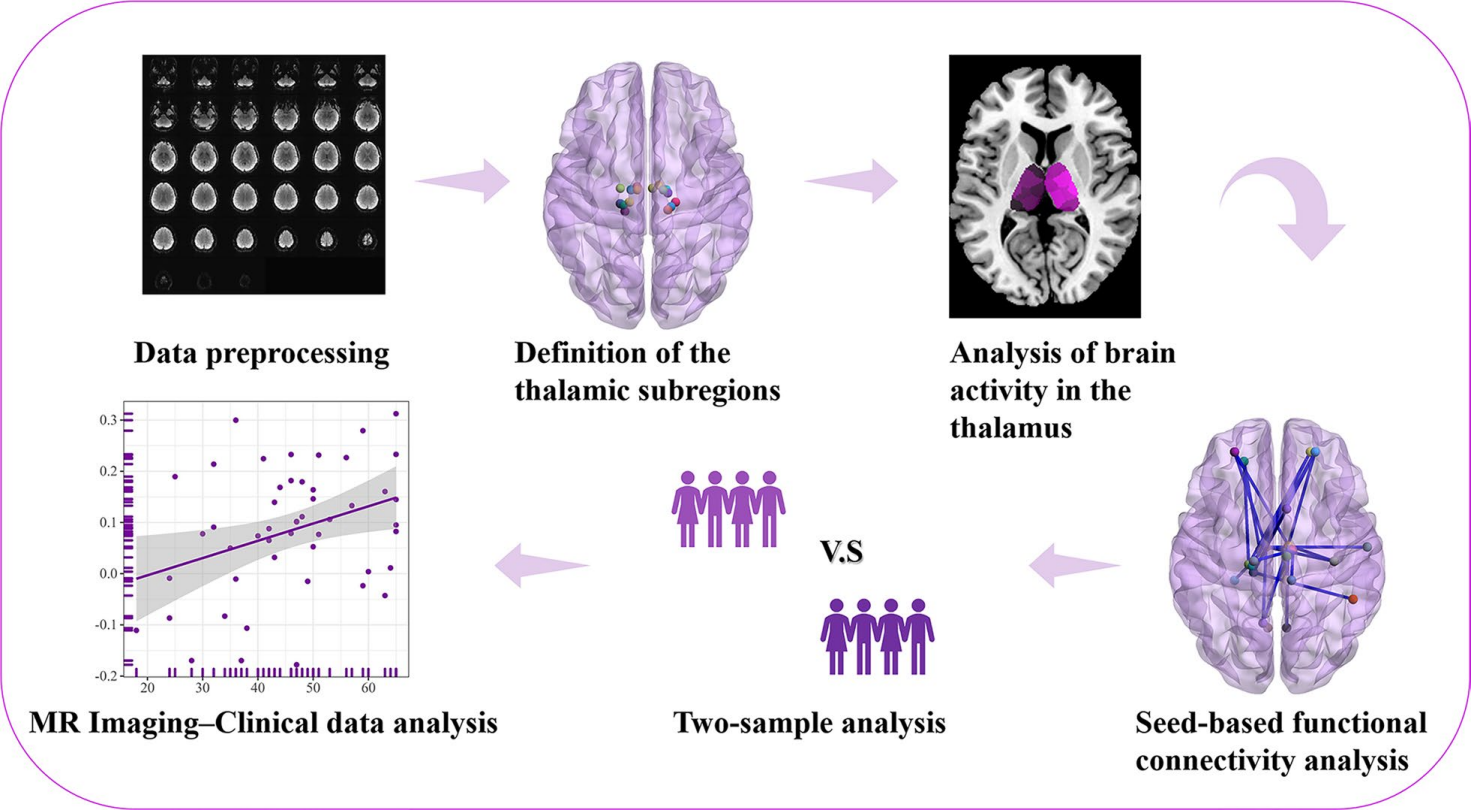
Data analysis flow chart

To determine the between-group differences in the fALFF and FC for the thalamic subregions, two-sample *t* test were performed, controlling for age, sex, education level, body mass index (BMI), GM, and mean FD values across the whole brain. Then, cluster-level false discovery rate (FDR) corrections were conducted, with a corrected threshold of *P* < 0.05. Pearson correlation analysis was used to estimate relationships in the clinical-MR imaging data. The clinical data includes: the duration of illness in patients with BN, the frequency of binge eating/purging per week, scores from the DEBQ, EDI-BN, EAT, BDI, and SAS questionnaires. The MR imaging data includes fALFF and FC values in the BN group. Statistical significance was set at *P* < 0.05, and FDR correction was conducted. The data processing steps are shown in Fig. 2.

## Results

### Demographic and clinical data

The demographic and clinical data were analyzed and are presented in Table 1. No significant differences were found between patients with BN and healthy controls (HCs) in terms of sex, age, education level, body mass index (BMI), and mean FD values. However, patients with BN exhibited higher scores on the DEBQ, EDI-BN, EAT, BDI, and SAS scales than HCs (*P* ≤ 0.001). In addition, the frequency of binge eating/purging per week and duration of illness are shown in Table 1 for patients with BN.

### Between-group differences in the thalamus fALFF

**Fig. 3:**
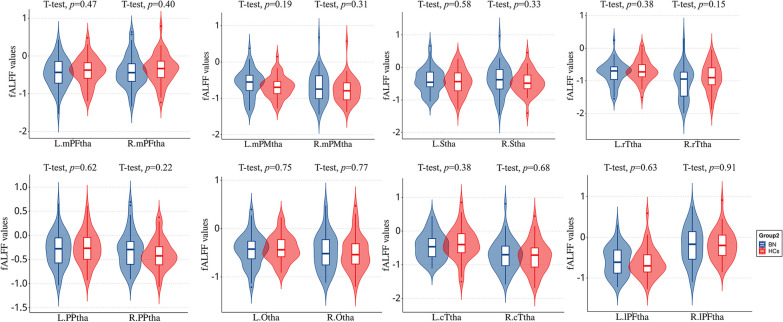
Comparison of the fractional amplitude of low-frequency fluctuation (fALFF) values in thalamic subregions between the BN and HC groups. No significant differences were found between the two groups after cluster-level FDR corrections were performed. mPFtha, medial pre-frontal thalamus; mPMtha, pre-motor thalamus; Stha, sensory thalamus; rTtha, rostral temporal thalamus; PPtha, posterior parietal thalamus; Otha, occipital thalamus; cTtha, caudal temporal thalamus; lPFtha, lateral pre-frontal thalamus; BN, bulimia nervosa; HCs, healthy controls

The fALFF values in all sixteen thalamic subregions did not differ significantly between the BN and HC groups (*P* < 0.05, cluster-level FDR-corrected) (Fig. 3).

### Between-group differences in the thalamus FC

#### LPFtha

Compared to HCs, patients with BN exhibited decreased FC between the left LPFtha and the bilateral prefrontal cortex, including the left middle frontal gyrus (MFG) and right superior frontal gyrus (SFG), compared to HCs (corrected threshold of cluster size > 77, *P* < 0.05).

#### PPtha

Compared to HCs, patients with BN showed decreased FC between the left PPtha and right MFG (corrected threshold of cluster size > 188, *P* < 0.05).

#### mPFtha

Compared to HCs, patients with BN showed decreased FC between the left mPFtha and prefrontal cortex (right MFG and left SFG) (corrected threshold of cluster size > 105, *p* < 0.05), right mPFtha and prefrontal cortex (right MFG and left SFG) and cingulate gyrus and Vermis_8 (corrected threshold of cluster size > 79, *P* < 0.05); increased FC between the left mPFtha and visual association regions, including the left fusiform gyrus (FFG) and left calcarine fissure (CAL), and primary sensorimotor cortex, including the right precentral gyrus (PreCG) and right postcentral gyrus (PoCG) (corrected threshold of cluster size > 65, *P* < 0.05); and increased FC between the right mPFtha and left CAL and right PreCG (corrected threshold of cluster size > 167, *P* < 0.05).

#### Stha

Compared to HCs, patients with BN showed decreased FC between the left Stha and prefrontal cortex (left MFG and right SFG) (corrected threshold of cluster size > 59, *P* < 0.05).

#### cTtha

Compared to HCs, patients with BN exhibited: decreased FC between the left cTtha and right MFG and right supplementary motor area (SMA) (corrected threshold of cluster size > 143, *p* < 0.05); increased FC between the left cTtha and left lingual gyrus (LING) and right superior temporal gyrus (STG) (corrected threshold of cluster size > 124, *p* < 0.05); and increased FC between the right cTtha and bilateral paracentral lobule (PCL) and right STG and left cerebellum_superior (cerebelum_4_5) (corrected threshold of cluster size > 63, *p* < 0.05).

#### Otha

Compared to HCs, patients with BN exhibited decreased FC between the left Otha and the right insula (INS), right MFG, right inferior parietal lobule (IPL) and right SMA (corrected threshold of cluster size > 59, *p* < 0.05, FDR).

**Fig. 4:**
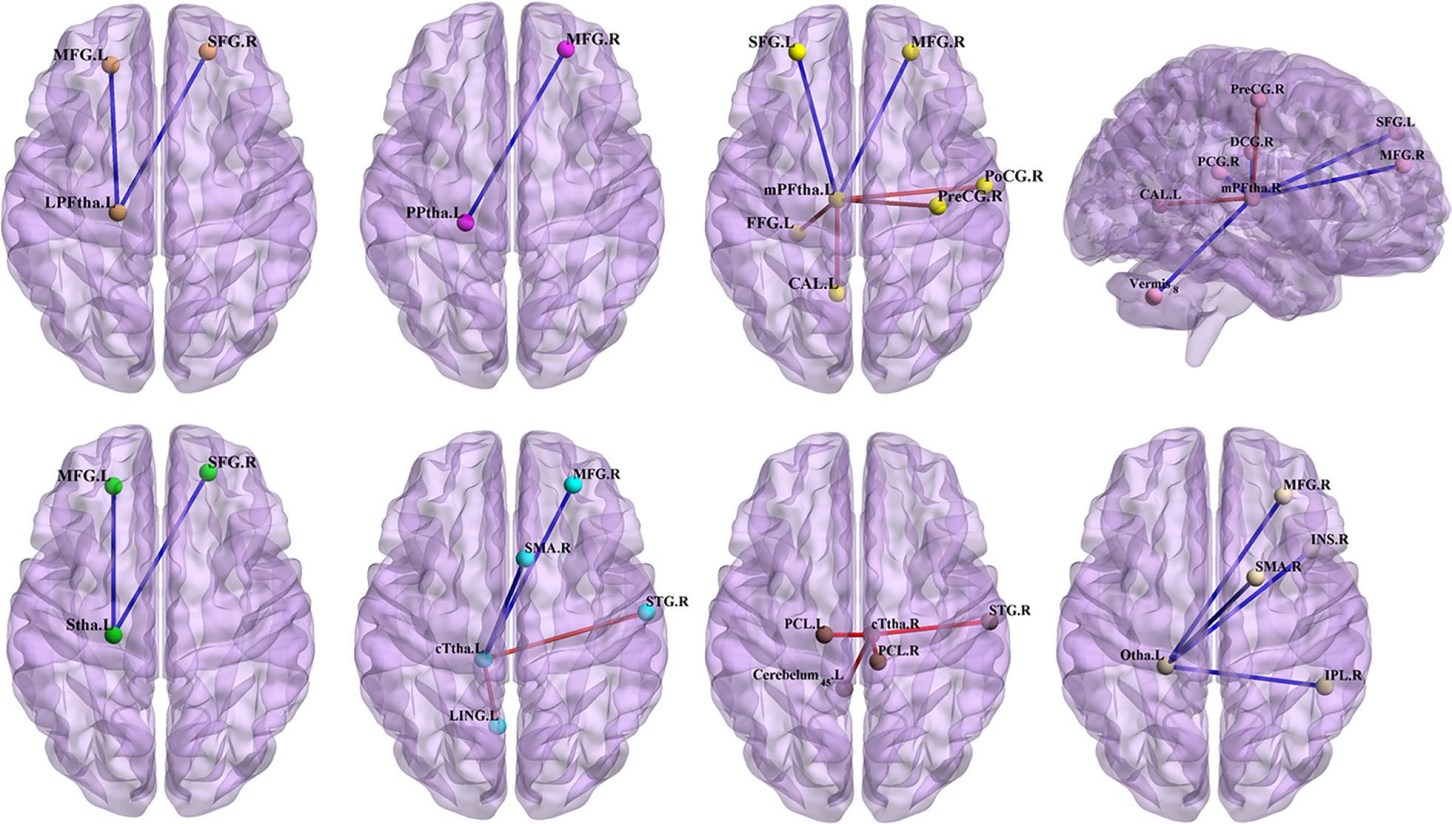
The functional connectivity (FC) of eight thalamic subregions was altered at the whole brain level in patients with bulimia nervosa (BN) compared to that of healthy controls (HCs). The red line indicates increased FC in the BN group, while the blue line indicates decreased FC in the BN group (*p* < 0.05, false discovery rate (FDR) correction at the cluster level). The brain regions represented in the plot include the lateral pre-frontal thalamus (LPFtha), posterior parietal thalamus (PPtha), medial pre-frontal thalamus (mPFtha), sensory thalamus (Stha), caudal temporal thalamus (cTtha), occipital thalamus (Otha), middle frontal gyrus (MFG), superior frontal gyrus (SFG), fusiform gyrus (FFG), calcarine fissure (CAL), postcentral gyrus (PoCG), precentral gyrus (PreCG), posterior cingulate gyrus (PCG), median cingulate and paracingulate gyri (DCG), supplementary motor area (SMA), lingual gyrus (LING), superior temporal gyrus (STG), cerebellum superior (Cerebelum_4_5), paracentral lobule (PCL), insula (INS), and inferior parietal lobule (IPL)

**Fig. 5:**
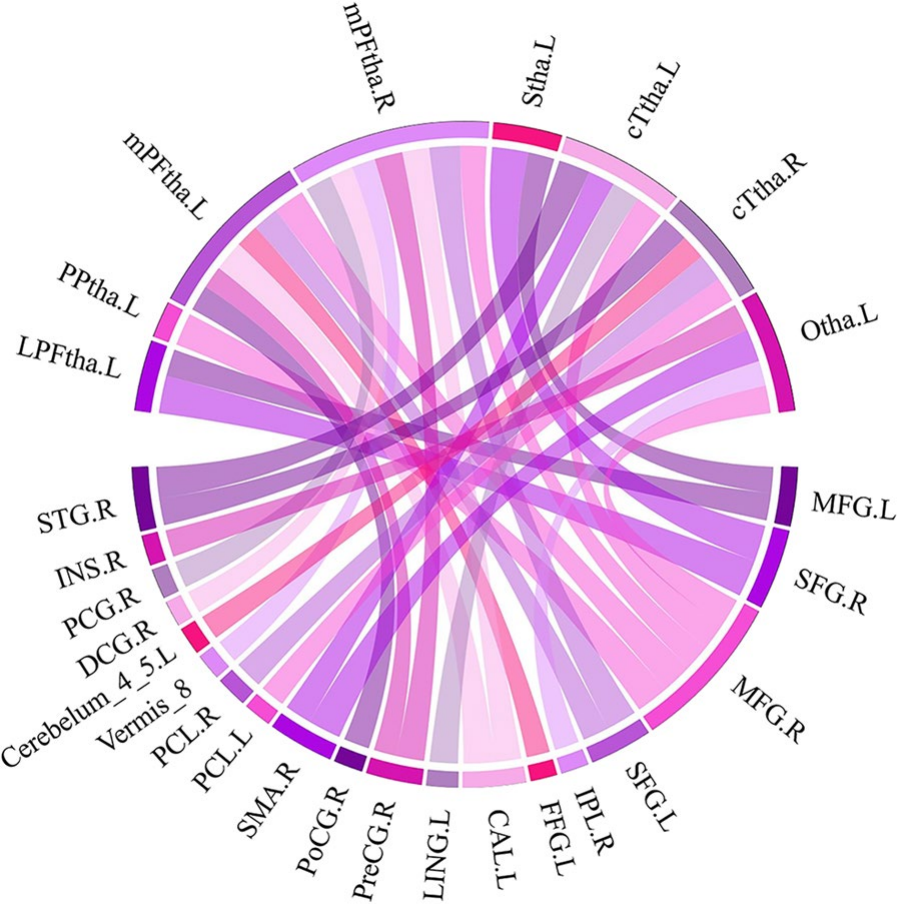
The "connectome ring" plot indicates altered interhemispheric functional connectivity (FC) patterns at the whole brain level in patients with bulimia nervosa (BN) compared to healthy controls (HCs). The FC differences between the BN and HC groups remained after cluster-level false discovery rate (FDR) corrections were performed (*P* < 0.05). The brain regions represented in the plot include the lateral pre-frontal thalamus (LPFtha), posterior parietal thalamus (PPtha), medial pre-frontal thalamus (mPFtha), sensory thalamus (Stha), caudal temporal thalamus (cTtha), occipital thalamus (Otha), middle frontal gyrus (MFG), superior frontal gyrus (SFG), fusiform gyrus (FFG), calcarine fissure (CAL), postcentral gyrus (PoCG), precentral gyrus (PreCG), posterior cingulate gyrus (PCG), median cingulate and paracingulate gyri (DCG), supplementary motor area (SMA), lingual gyrus (LING), superior temporal gyrus (STG), cerebellum superior (Cerebelum_4_5), paracentral lobule (PCL), insula (INS), and inferior parietal lobule (IPL)

**Fig. 6:**
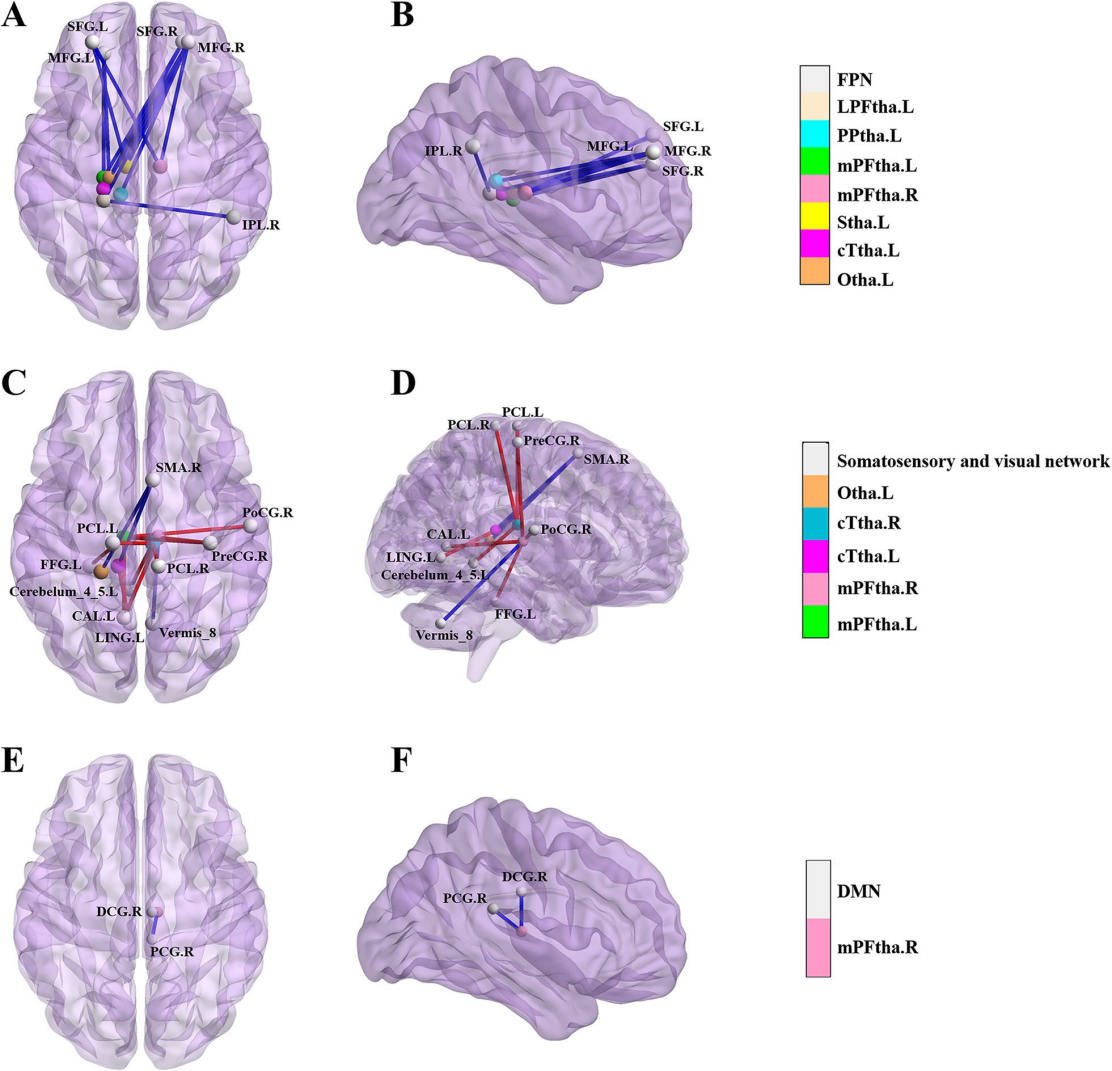
A map of the brain network, illustrating the functional connectivity (FC) between various thalamic subregions and brain regions located within the frontoparietal, somatosensory, visual, and default mode networks. In bulimia nervosa (BN) patients, the FC between the thalamic subregions, including the lateral pre-frontal thalamus (LPFtha), posterior parietal thalamus (PPtha), medial pre-frontal thalamus (mPFtha), sensory thalamus (Stha), caudal temporal thalamus (cTtha), and occipital thalamus (Otha), and the brain regions in the frontoparietal network were observed to be altered compared to that in healthy controls (HCs) (**A**, **B**). The FC between thalamic subregions, including mPFtha, cTtha, and Otha, and brain regions in the somatosensory and visual networks was observed to be altered in BN patients compared with that in HCs (**C**, **D**). The FC between the mPFtha and brain regions within the default mode network was observed to be altered in BN patients compared with that in HCs (**E**, **F**). The blue line denotes decreased FC, while the red line indicates increased FC

All the above results were corrected with FDR correction at the cluster-level *p* < 0.05. Further details are included in Table 2, Figs. 4, 5, and 6.

**Table 2:** Regions showing group differences (BN vs. HCs) in thalamus connectivity

Seed	Region with altered FC	Peak MNI coordinate			Cluster size	Peak intensity
		X	Y	Z		
Left LPFtha	Left MFG	− 21	42	30	77	− 4.481
	Right SFG	21	48	24	160	− 6.157
Left PPtha	Right MFG	24	48	30	188	− 5.399
Left mPFtha	Right MFG	24	48	24	259	− 5.181
	Left SFG	− 27	48	39	105	− 4.322
	Left FFG	− 27	− 33	− 24	65	3.874
	Left CAL	− 9	− 60	6	303	5.051
	Right PoCG	57	− 12	15	181	4.264
	Right PreCG	36	− 21	63	96	3.739
Right mPFtha	Vermis_8	6	− 63	− 36	86	− 3.909
	Right PCG	6	− 33	21	98	− 4.449
	Right MFG	27	51	24	114	− 4.318
	Left SFG	− 27	48	39	199	− 5.084
	Right DCG	0	− 18	30	79	− 3.800
	Left CAL	− 9	− 60	6	167	4.442
	Right PreCG	− 3	− 30	57	766	4.635
Left Stha	Left MFG	− 21	42	30	59	− 4.347
	Right SFG	21	48	24	124	− 5.966
Left cTtha	Right MFG	27	45	36	143	− 4.612
	Right SMA	6	12	57	154	− 4.410
	Left LING	− 6	− 63	0	190	4.660
	Right STG	69	− 12	9	126	5.191
Right cTtha	Left Cerebelum_4_5	− 6	− 45	− 3	113	4.094
	Right STG	69	− 15	9	76	4.408
	Right PCL	9	− 33	72	146	4.635
	Left PCL	− 15	− 21	78	63	4.104
Left Otha	Right INS	42	15	0	68	− 4.466
	Right MFG	30	39	36	183	− 4.563
	Right IPL	48	− 45	33	59	− 4.105
	Right SMA	18	3	75	248	− 5.459

*LPFtha* lateral pre-frontal thalamus, *PPtha* posterior parietal thalamus, *mPFtha* medial pre-frontal thalamus, *Stha* sensory thalamus, *cTtha* caudal temporal thalamus, *Otha* occipital thalamus, *MFG* middle frontal gyrus, *SFG* superior frontal gyrus, *FFG* fusiform gyrus, *CAL* calcarine fissure, *PoCG* Postcentral gyrus, *PreCG* precentral gyrus, *PCG* posterior cingulate gyrus, *DCG* median cingulate and paracingulate gyri, *SMA* supplementary motor area, *LING* Lingual gyrus, *STG* superior temporal gyrus, *Cerebelum_4_5* cerebellum superior, *PCL* paracentral lobule, *INS* Insula, *IPL* inferior parietal lobule, *MNI* Montreal Neurological Institute, *BN* bulimia nervosa, *HCs* healthy controls; All results were corrected by cluster-level false discovery rate (FDR) correction with a corrected threshold of *P* < 0.05

### MR imaging data and clinical relationships

**Fig. 7:**
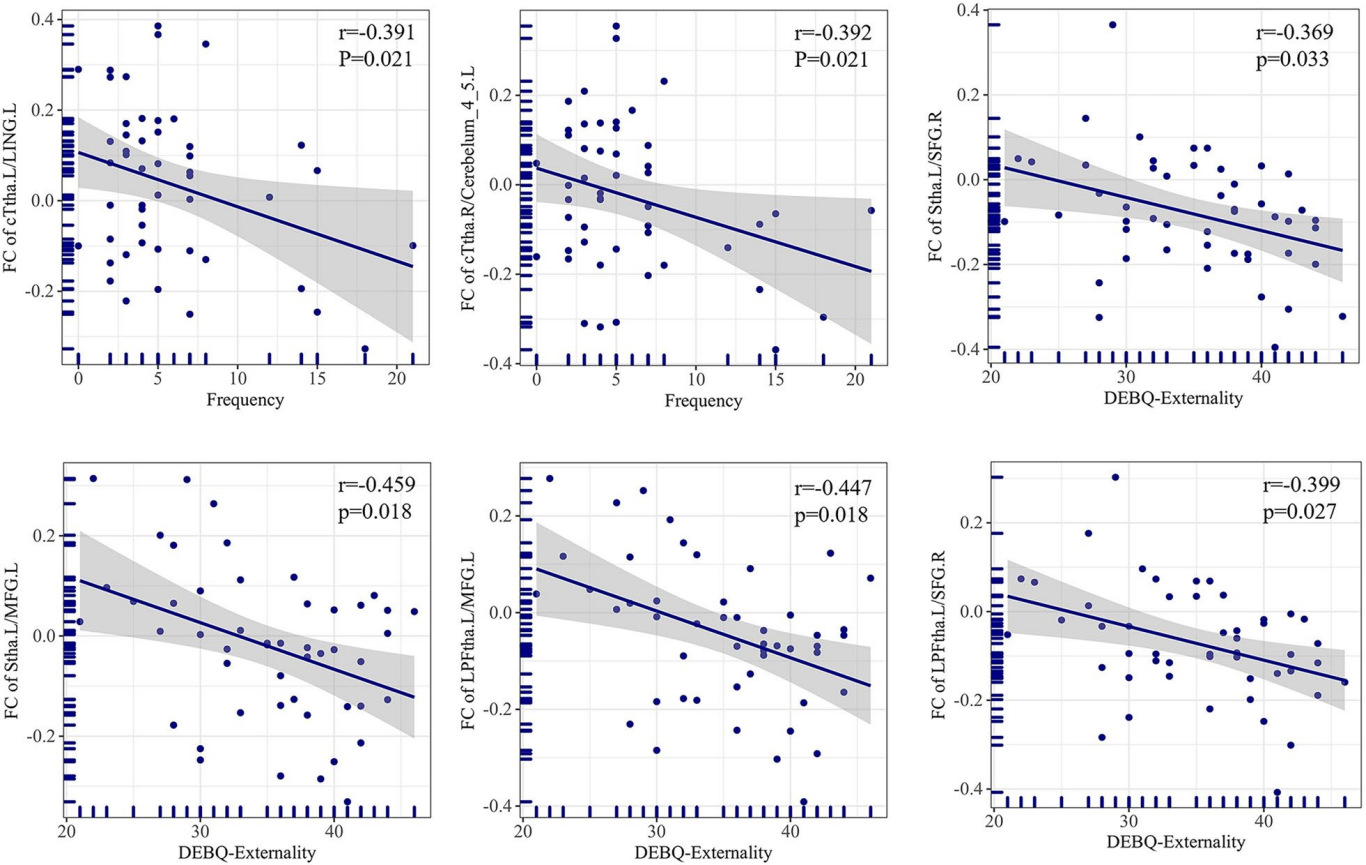
Significant correlations were observed between the functional connectivity (FC) of thalamic nuclei and behavioral measures in patients with bulimia nervosa (BN). Negative correlations were found between the frequency of binge/purge eating episodes per week and the FC of the left caudal temporal thalamus (cTtha.L) and left lingual gyrus (LING.L), as well as the FC of the right cTtha/left Cerebelum_4_5. Negative correlations were observed between the scores on the Dutch Eating Behavior Questionnaire-Externality (DEBQ-Externality) test and the FC between the thalamic nuclei and prefrontal cortex, including the right superior frontal gyrus (SFG) and left middle frontal gyrus (MFG)

In BN patients, the FC between the left cTtha and left LING and between the right cTtha and left cerebelum_4_5 showed negative correlations with the frequency of binge eating/purging times per week. The FC between the thalamus subregions (Stha, LPFtha) and prefrontal cortex (MFG, SFG) was negatively correlated with DEBQ-Externality scores (Table 3; Fig. 7). All the above results were corrected with FDR correction at the cluster-level *p* < 0.05.

**Table 3:** The correlation between clinical characteristics and MR-imaging data

Clinical characteristics	MR-imaging data	Coefficient	*P* value
Frequency	FC of cTtha.L/LING.L	− 0.391	0.021*
	FC of cTtha.R/Cerebelum_4_5. L	− 0.392	0.021*
DEBQ-externality	FC of Stha.L/SFG.R	− 0.369	0.033*
	FC of Stha.L/ MFG.L	− 0.459	0.018*
	FC of LPFtha.L/ MFG.L	− 0.447	0.018*
	FC of LPFtha.L/ SFG.R	− 0.399	0.027*

*Statistical significance (FDR correction); *Frequency* the frequency of binge eating/purging per week, *DEBQ* Dutch Eating Behavior Questionnaire, *Stha* sensory thalamus, *LPFtha* lateral pre-frontal thalamus, *LING* Lingual gyrus, *Cerebelum_4_5* cerebellum superior, *MFG* middle frontal gyrus, *SFG* superior frontal gyrus

## Discussion

In this study, fALFF and seed-based FC analyses were used to investigate thalamic neural activity and FC throughout the brain in both the BN and HC groups, with thalamic nuclei used as seed points. The results showed no significant differences in neural activity in the thalamus between the two groups. However, in contrast to HCs, the patients in the BN group showed significant changes in FC between thalamic nuclei and other brain regions, including the central components of the frontoparietal network (FPN), default mode network (DMN), somatosensory and visual network. This observation may imply that among individuals with BN, local neural activity within the thalamus remains relatively stable. In contrast, notable changes in thalamic regulation occur within the functional network at the whole-brain level. Consequently, it becomes imperative to shift our attention towards the thalamus's pivotal role in modulating the comprehensive functional network across the entire brain, rather than solely concentrating on localized functional activity changes within the thalamus. Additionally, we observed significant changes in FC between the thalamic subregion and core brain regions within the FPN, somatosensory and visual network through correlation analysis. These alterations were closely associated with clinical symptoms in patients with BN, including disordered eating behavior and the severity of illness. Therefore, these findings suggest that changes in FC between the thalamic subregion and the FPN, somatosensory and visual network may play a crucial role in regulating abnormal eating behaviors in BN. The thalamus, serving as a central hub in the regulation of feeding behavior and functioning as a higher-order relay station relay station for neural processes, may offer further insights into the neurobiological patterns underlying eating disturbances in individuals with BN through alterations in its FC. Simultaneously, the core brain regions within the FPN, somatosensory and visual network hold promise as potential targets for future neuromodulation interventions for BN, laying a solid theoretical foundation for enhancing clinical outcomes in therapy. Further clarification of these patterns is expected to enhance our comprehension of the neural regulatory mechanisms in BN, thereby providing additional theoretical support for subsequent research related to alterations in core brain regions associated with appetite regulation.

The thalamus is responsible for conveying various types of information, such as cognition, execution, interception, emotion, and vision function, through different nuclei in parallel pathways [11, 30]. In the present study, although the regional functional activity of thalamic subregions did not differ significantly between individuals with BN and HCs, significant alterations in FC were observed at the whole-brain level, with certain subregions acting as seed nodes. These subregions included the left lPFtha, left PPtha, bilateral mPFtha, left Stha, bilateral cTtha and left Otha. Thus, while the functional activity of the thalamus itself may not be abnormally altered at rest in individuals with BN, the pathways through which other brain regions communicate with the thalamus may be altered.

Specifically, we found that the bilateral MFG, bilateral SFG and right IPL, which are all central components of the FPN [31, 32], had significantly decreased FC with thalamic nuclei in BN patients compared to that in HCs. The FPN is regarded as a top-down processing system that is implicated in various executive functions, including cognition, attention and emotion [32–34]. Previous studies have shown that functional and anatomical alterations in the FPN are associated with BN. [9, 35, 36]. For example, abnormal cortical thickness and structural connectivity in FPN regions have been linked to cognitive impairment in individuals with BN, and changes in FC within the FPN have been linked to symptom improvement in BN patients [35, 36]. Lan et al. [9] found that reduced inter-modular connections between the FPN and cerebellum, as well as altered nodal properties in the prefrontal cortex and IPL, were associated with clinical variables such as interoceptive awareness, maturity fears, and bulimia symptoms. Based on the previous literature, our study has revealed further alterations in the FC between the thalamus and brain regions in the FPN. Furthermore, evidence from functional neuroimaging studies suggests that the STG plays a role in social cognition, emotion, and top-down attentional control, similar to the functional activity of the FPN [37, 38]. For instance, Balodis et al. [39] used an fMRI Stroop task to investigate the correlation between neural activity and inhibitory control. They observed diminished activity in the superior temporal areas in individuals with binge-eating compared to individuals without binge-eating. This implies that alterations in the functional activity of the STG may be associated with inhibitory control deficits among individuals with BN [39].

Overall, our results suggest that the altered FC between the thalamus and brain regions in the prefrontal, inferior parietal, and superior temporal cortices may be related to cognition, impulsivity, and attention function in individuals with BN. Furthermore, the FC between the prefrontal cortex (MFG, SFG) and thalamic nuclei appears to be related to external eating behaviors, indicating a neurobiological correlate between the altered functional activity of the FPN and abnormal eating behaviors in individuals with BN. However, future studies using task-based designs are needed to confirm these associations.

In addition, our study found that compared to HCs, individuals with BN showed significantly altered FC between thalamic nuclei and central components of the somatosensory and visual networks, such as the PoCG, PreCG, SMA, PCL, cerebellum, FFG, CAL, and LING [40, 41]. The somatosensory and visual networks integrate sensorimotor and visual information to construct body image perceptions, and a distorted representation of one's own body is a diagnostic criterion for BN [40, 42]. Previous studies have suggested that individuals with BN overestimate their body size [43]. Wang et al. [8]found changes in FC across sensorimotor and visual regions with the striatum, and Lavagnino et al. [44] revealed reduced FC across the somatosensory network and visual cortex in patients with BN, which further supports our findings.​ According to the results of our study, the altered FC between the thalamic nuclei and these sensorimotor and visual regions may be related to negative self-evaluation of body image in individuals with BN. Moreover, we observed that the altered FC between the thalamic nuclei and LING and cerebellum was correlated with the frequency of BN symptoms. These results may suggest that somatosensory and visuospatial disturbances are correlated with illness severity in BN patients.

The PCC is part of the DMN, which is closely linked to self-referential mental activity, particularly during rest. Numerous studies have shown that the DCG is associated with cognitive function, possibly as part of the default network [45–47]. The DMN is highly correlated with cognitive function [48], and failure to deactivate the DMN may lead to constant preoccupation with food or body image-related thoughts in individuals with BN [49]. The insula, which is often referred to as the salience network, integrates neural systems involved in processing external sensory information and interoceptive awareness [50]. Previous research has highlighted the insula's importance in processing food stimuli, with spatial brain patterns in the right insular cortex achieving the greatest diagnostic accuracy for separating BN patients from normal-weight controls [51]. In a study using a well-validated cued breathing load task, Berner et al. found that individuals in remission from BN showed increased activation in the insula, PCC, and middle cingulate gyrus during breathing load anticipation, suggesting altered anticipation and experience of body sensations in individuals with eating disorders [52]. Our findings add to increasing evidence that functional alterations in the insula and cingulate gyrus are highly related to BN [51–55]. In addition, according to our results, the altered FC between thalamic nuclei and the cingulate gyrus and insula may be related to the altered interoceptive awareness of body sensations and processing of external sensory information (i.e., body image and food stimuli processing) in individuals with BN.

Individuals with anorexia nervosa (AN) often display an intense preoccupation with their own body size and weight, and they may also experience a profound distortion in their perception of body image, often incorrectly believing themselves to be overweight despite having a dangerously low actual weight [15]. Hence, it is reasonable to hypothesize that there may be some degree of neurophysiological similarities in the self-assessment of body shape and weight between BN and AN, particularly the binge-purging subtype of AN (AN binge-purging subtype). The thalamus plays a crucial role in transmitting sensory signals and holds significant importance in the development and maintenance of AN [56]. Several studies have extensively investigated the alterations in thalamic white matter microstructure in individuals with AN. Specifically, changes in thalamic white matter microstructure may lead to disruptions in self-regulation functions among patients with AN [57] and could also contribute to alterations in their visual processing of food [58]. Furthermore, research has indicated a potential association between structural changes in the thalamus and the body weight of AN patients [59]. Despite clinical similarities between AN and BN patients, differences in body weight hold crucial diagnostic significance. Therefore, building upon the research on thalamic white matter microstructure in AN patients and our investigation into thalamic functional activity in BN, further exploration of how thalamic structure influences cognitive regulation, visual processing, and body weight regulation in BN patients will provide robust support for a deeper understanding of the mechanisms underlying eating disorders.

This study has several limitations that must be considered. First, the sample size of this study is moderate, and the sample size should be increased in future studies to enhance the reliability of the findings. Second, a study on thalamic volume in patients with AN has shown that during the acute phase of the disorder, there is a reduction in both thalamic volume and complexity [59]. Collantoni et al. [59]. speculate on the substantial influence of malnutrition-related mechanisms on the structure of the thalamus in individuals with AN. Both AN and BN are classified as eating disorders. Patients with AN typically exhibit a body weight significantly lower than that of healthy individuals, while individuals with BN tend to fall within the normal weight or overweight range [15]. Consequently, additional research is required to explore thalamic structural changes in individuals with BN and to further investigate the relationship between malnutrition-related mechanisms and alterations in thalamic structure. Third, additional studies should be performed to explore causal relationships between thalamic network changes and BN and to examine potential interventions targeting thalamic circuits to improve treatment outcomes in BN patients. Fourth, in future research, we intend to collect data on the precise temporal intervals between the last binge/purge episode, other compensatory behaviors, and the timing of the MRI scans. This enhanced data collection will facilitate a more in-depth investigation into the interplay between neural activity in individuals diagnosed with BN and their patterns of eating behavior.

## Conclusions

In this study, the resting-state functional activity of the thalamus was investigated in patients with BN in this study. Our findings indicate that the FC between thalamic nuclei and other brain regions is altered throughout the whole brain. These changes were primarily observed within the FPN, DMN, somatosensory and visual network. Importantly, the changes in FC between thalamic nuclei and brain regions within the FPN, somatosensory and visual networks were significantly associated with disordered eating behavior and the severity of illness in BN patients. Although we did not observe significant differences in regional neural activity in the thalamus between the BN and HC groups, our results suggest that disrupted thalamic connectivity may contribute to the pathophysiology of BN. The above findings may help identify the neural mechanisms underlying disordered eating behavior and the severity of illness in BN, and may also suggest potential targets for future neuromodulation interventions.

### Supplementary Information

**Additional file 1.** The MNI coordinates of the 16 subregions of the thalamus in the HBA atlas.

## Data Availability

The data in the current study are available from the corresponding author on reasonable request.

## Abbreviations

3D-MPRAGE: Three-dimensional magnetization-prepared rapid gradient-echo
ALFF: Amplitude of low-frequency fluctuation
AN: Anorexia nervosa
BDI-II: Beck depression inventory-II
BMI: Body mass index
BN: Bulimia nervosa
CAL: Calcarine fissure
CAT12: Computational Anatomy Toolbox 12
Cerebelum_4_5: Cerebellum superior
CSF: Cerebrospinal fluid
cTtha: Caudal temporal thalamus
DARTE: Diffeomorphic anatomical registration through exponentiated lie algebra
DCG: Median cingulate and paracingulate gyri
DEBQ: Dutch Eating Behavior Questionnaire
DMN: Default mode network
DPABI: Data Processing and Analysis for Brain Imaging
DSM-5: Diagnostic and statistical manual of mental disorders, fifth edition
EAT: Eating attitude test
EDI-1: Eating disorder inventory-1
EPI: Echo-planar imaging
FA: Flip angle
fALFF: The fractional amplitude of low-frequency fluctuation
FC: Functional connectivity
FD: Framewise displacement
FDR: False discovery rate
FFG: Fusiform gyrus
FOV: Field of view
FPN: Frontoparietal network
FWHM: Full-width half-maximum
GM: Gray matter
HBA: Human brainnetome atlas
HCs: Healthy controls
INS: Insula
IPL: Inferior parietal lobule
LING: Lingual gyrus
lPFtha: Lateral pre-frontal thalamus
MFG: Middle frontal gyrus
MINI: Mini-International Neuropsychiatric Interview
MNI: Montreal Neurological Institute
mPFtha: Medial pre-frontal thalamus
mPMtha: Pre-motor thalamus
Otha: Occipital thalamus
PCG: Posterior cingulate gyrus
PCL: Paracentral lobule
PoCG: Postcentral gyrus
PPtha: Posterior parietal thalamus
PreCG: Precentral gyrus
rTtha: Rostral temporal thalamus
SAS: Self-rating Anxiety Scale
SFG: Superior frontal gyrus
SMA: Supplementary motor area
SPM12: Statistical Parametric Mapping 12
STG: Superior temporal gyrus
Stha: Sensory thalamus
TE: Echo time
TR: Repetition time
WM: White matter
