# Supplementary material for: Exploring the thalamus: a crucial hub for brain function and communication in patients with bulimia nervosa
Source: J Eat Disord. 2023 Nov 20;11:207. doi: 10.1186/s40337-023-00933-6 (PMC10662785; doi:10.1186/s40337-023-00933-6)
Supplement: Supplementary file 1 — Additional file 1. The MNI coordinates of the 16 subregions of the thalamus in the HBA atlas. [file 40337_2023_933_MOESM1_ESM.docx]

The thalamus was defined based on the Human Brainnetome Atlas, and the thalamus was further divided in sixteen subregions on the bilateral sides as follows: left mPFtha (x = -7, y = -12, z = 5), right mPFtha (x = 7, y = -11, z = 6), left mPMtha (x = -18, y = -13, z = 3), right mPMtha (x = 12, y = -14, z = 1), left Stha (x = -18, y = -23, z = 4), right Stha (x = 18, y = -22, z = 3), left rTtha (x = -7, y = -14, z = 7), right rTtha (x = 3, y = -13, z = 5), left PPtha (x = -16, y = -24, z = 6), right PPtha (x = 15, y =-25, z = 6), left Otha (x = -15, y = -28, z = 4), right Otha (x = 13, y = -27, z = 8), left cTtha (x = -12, y = -22, z = 13), right cTtha (x = 10, y = -14, z = 14),left lPFtha (x = -11, y = -14, z = 2), and right lPFtha (x = 13, y = -16, z = 7).
